# Outcomes of perioperative nursing practices performed by operating room nurses: a scoping review

**DOI:** 10.1186/s12912-026-05104-x

**Published:** 2026-08-03

**Authors:** Ayano Ando, Runa Tokunaga, Makoto Kaneko

**Affiliations:** 1 Institute of Inclusive Community, Sophia University, 7- 1 Kioi-cho, Chiyoda-ku, Tokyo, 102-8554 Japan; 2 Department of Nursing, Faculty of Human Sciences, Sophia University, 7-1 Kioi-cho, Chiyoda-ku, Tokyo, 102-8554 Japan; 3 Department of Health Data Science, Yokohama City University, 22-2, Seto, Kanazawa-ku, Yokohama, Kanagawa 236-0027 Japan; 4 Sagamihara Endowed Chair in Comprehensive Community Medicine, Kitasato University School of Medicine, 1-15-1, Kitazato, Minami-ku, Sagamihara-shi Kanagawa, 252-0373 Japan; 5 Present Address: Department of Nursing, Faculty of Medical Technology, Teikyo University, 2-11-1 Kaga, Itabashi-ku, Tokyo, 173-8605 Japan; 6 Present Address: Department of General Practice, Kitasato University School of Medicine, 1-15-1 Kitazato, Minami-ku, Sagamihara-shi, Kanagawa, 252-0373 Japan

**Keywords:** Operating room nurses, Perioperative nursing, Perioperative outcomes, Review

## Abstract

**Background:**

The importance of perioperative nursing is increasingly being recognized; however, how operating room nurses contribute to patient outcomes remains underexplored, because nursing roles are often not distinguished. This study aims to map outcomes associated with perioperative nursing practices performed by operating room nurses, focusing on outcome types, measurement methods, and timing of evaluation. This review addresses the question of what outcomes of perioperative nursing practices performed by operating room nurses have been reported.

**Methods:**

This scoping review follows Arksey and O’Malley’s framework, incorporating refinements by Levac et al., and adheres to the JBI guidelines. A comprehensive literature search was conducted using PubMed, CINAHL Plus, and EMBASE, without restrictions on publication year, targeting peer-reviewed English-language articles published up to May 28, 2025, excluding case reports, reviews, and non-peer-reviewed studies. Studies reporting outcomes related to perioperative nursing practices by operating room nurses were included. Data were extracted and descriptively synthesized, with outcomes categorized by perioperative phase.

**Results:**

Twenty-four studies published between 1991 and 2025 were included, with randomized controlled trials being the most common (*n* = 10). Reported outcomes spanned the entire perioperative period. Preoperative outcomes primarily involved psychological indicators, intraoperative outcomes focused on physiological stability, and postoperative outcomes included pain, recovery, complications, patient satisfaction, and quality of life. Outcome definitions, measurement methods, and evaluation time points varied across studies. Overall, the mapped outcomes varied widely across the perioperative phases, highlighting marked heterogeneity and a lack of standardization.

**Conclusions:**

This scoping review descriptively synthesizes the outcomes used to evaluate perioperative nursing practices performed by operating room nurses across the perioperative phases. Diverse outcomes were reported across studies, reflecting the multifaceted nature of operating room nursing practice. Developing standardized, nursing-specific core outcome sets may improve consistency in future research and support evidence-based practice and education.

**Review registration:**

This review protocol was prospectively registered with the UMIN Clinical Trials Registry (UMIN-CTR; ID: UMIN000058114; Registration date: June 7, 2025).

**Supplementary Information:**

The online version contains supplementary material available at https://doi.org/10.1186/s12912-026-05104-x.

## Background

The quality of perioperative nursing has been associated with surgical safety, postoperative recovery, and patient satisfaction [1]. Recent advances in medical technology have resulted in increasingly sophisticated surgical procedures, necessitating skilled and adaptive nursing practice. The perioperative period is divided into three phases—preoperative, intraoperative, and postoperative—during which patients undergo significant physical and psychological changes [2, 3]. The preoperative phase extends from the time of surgical decision to anesthesia induction; the intraoperative phase covers anesthesia induction through surgery completion and emergence; the postoperative phase spans from post-surgery through post-anesthesia care unit management until discharge. Operating room nurses are uniquely positioned to support patients across all perioperative phases, particularly during the intraoperative period [4]. Their responsibilities include providing preoperative explanations and psychological support, ensuring intraoperative patient safety, and facilitating postoperative information sharing and continuity of care [4].

The Association of periOperative Registered Nurses (AORN) describes the role of perioperative registered nurses as including the planning, implementation, and evaluation of nursing care from preoperative preparation through intraoperative care to postoperative recovery [3]. This description highlights that perioperative nursing addresses patients’ physiological, psychological, sociocultural, and spiritual responses [3]. Therefore, operating room nursing practice extends beyond technical tasks to enhance the overall quality of care and the patient’s experience. McGarvey et al. [5] noted that operating room nurses’ responsibilities have expanded owing to technological advances, encompassing infection prevention, equipment management, patient monitoring, and interprofessional collaboration. This evolution underscores the operating room nurse as an important contributor to the quality of perioperative care. Recent studies have indicated that preoperative outpatient consultations or home visits by operating room nurses, involving information collection and provision, have been associated with reduced patient anxiety and improved postoperative pain scores [6]. For instance, when nurses provide tailored information and engage in active listening preoperatively, patients are more likely to adopt a proactive approach to surgery and recovery, consistent with evidence that preoperative education enhances adherence to postoperative recovery protocols [7].

Despite growing recognition of the importance of perioperative nursing, the specific contributions of operating room nurses to patient outcomes remain insufficiently synthesized. Existing research often does not distinguish between different nursing roles, making it difficult to attribute specific patient outcomes to operating room nursing practices. Although individual studies have reported a range of favourable findings, the specific outcome domains, measurement instruments, and assessment time points used to evaluate this care have not been systematically mapped, leaving it unclear where evidence is concentrated and where it remains lacking. Therefore, a comprehensive mapping of interventions and associated outcomes is needed to clarify how these practices are evaluated and to inform future research, clinical practice, and education.

This scoping review aims to map outcomes associated with perioperative nursing practices conducted by operating room nurses, focusing on outcome types, measurement methods, and evaluation timing.

## Methods

### Design

A scoping review was conducted using the framework developed by Arksey and O’Malley [8] and refined by Levac et al. [9] and the JBI [10]. The review followed the procedures outlined in the Joanna Briggs Institute Manual for Evidence Synthesis [10]. The review protocol was prospectively registered with the UMIN Clinical Trials Registry (UMIN-CTR; ID: UMIN000058114; Registration date: June 7, 2025).

### Search methods

The literature search aligned with the research questions based on the Population, Concept, Context framework and was conducted with the assistance of a medical librarian. The databases searched included PubMed, CINAHL Plus, and EMBASE. To comprehensively capture the full range of outcomes reported in the literature, no publication year restrictions were applied, and eligible were articles published up to May 28, 2025, with the earliest study published in 1991. An unrestricted search strategy was deemed necessary to capture the full range of evidence on outcomes linked to perioperative nursing practices by operating room nurses.

The Population, Concept, Context framework was defined as follows:

- Population: Operating room nurses providing intraoperative care, including scrub, circulating, and other nursing roles within the operating room, such as anesthesia-related roles where applicable.
- Concept: nursing practices.
- Context: perioperative period, from the decision to undergo surgery to hospital discharge, in both inpatient and outpatient settings.

Based on this framework, the review addresses the following research questions:

What outcomes of perioperative nursing practices performed by operating room nurses have been reported? What measurement methods and assessment time points have been used for these outcomes? The search strategy integrated controlled vocabulary (MeSH, CINAHL Headings, EMTREE) with free-text terms in titles and abstracts, using the following keywords:

(“operating room nursing” OR “perioperative nursing” OR “surgical nursing” OR “medical surgical nursing”) AND (“preoperative care” OR “preoperative period” OR “postoperative care” OR “postoperative period”) (Additional file 1).

The database search covered all records from inception to May 28, 2025.

### Inclusion and/or exclusion criteria

The inclusion criteria were:

1. Studies reporting patient-, clinical-, or care-related outcomes associated with perioperative nursing practices provided by operating room nurses across the preoperative, intraoperative, and postoperative phases.
2. Peer-reviewed articles published in English.

The exclusion criteria were:

1. Publications lacking an academic research design, such as case reports, discussion papers, commentaries, editorials, letters, expert opinions, news articles, conference abstracts, reviews without full text, or non-peer-reviewed publications.
2. Secondary sources (e.g., review articles).

### Screening and eligibility assessments

All retrieved records were managed in a reference system, and duplicates were removed before screening. Two independent reviewers (AA and RT) evaluated the titles and abstracts against the eligibility criteria. The full texts of potentially relevant studies were assessed, and studies reporting outcomes of operating room nurses’ perioperative practices were included. The study selection and reporting of this scoping review were conducted in accordance with the PRISMA Extension for Scoping Reviews checklist [11].

Consistent with scoping review methodology, no formal critical appraisal of study quality was conducted. Screening and eligibility assessments were conducted by three reviewers using the Rayyan QCRI online platform [12]. Any disagreements between the primary reviewers were first discussed, and unresolved discrepancies were adjudicated by a third reviewer until consensus was reached. Additionally, backward citation searching (screening of the reference lists of all included studies) was performed to identify further relevant studies. The reference lists of the 21 studies included from the database search were screened, yielding 28 additional records for eligibility assessment; of these, 3 studies met the inclusion criteria and were added, resulting in a total of 24 included studies.

### Data abstraction

Data from the selected studies were extracted and organized by author, publication year, country, study objectives, research design, methods, and main findings. The extracted data were systematically classified according to the perioperative phase—preoperative, intraoperative, and postoperative—to capture the temporal variations in nursing interventions and outcomes.

### Synthesis

Within each phase, data were further organized by nursing practices, study populations, outcome types, measurement methods, and the timing of outcome assessments. This phase-based framework enabled a structured comparison of the manner in which operating room nurses’ practices influenced different aspects of patient care and recovery throughout the perioperative continuum.

### Ethical considerations

Ethical approval was not required because this study uses publicly available data.

### Declaration of generative AI and AI-assisted technologies in the manuscript preparation process

During the preparation of this manuscript, the authors used ChatGPT (OpenAI, GPT-5) to assist with text organization, summarization of background information, and refinement of language. All AI-generated content was carefully reviewed, edited, and verified by the authors, who take full responsibility for the accuracy, validity, and interpretation of the manuscript.

## Results

**Fig. 1:**
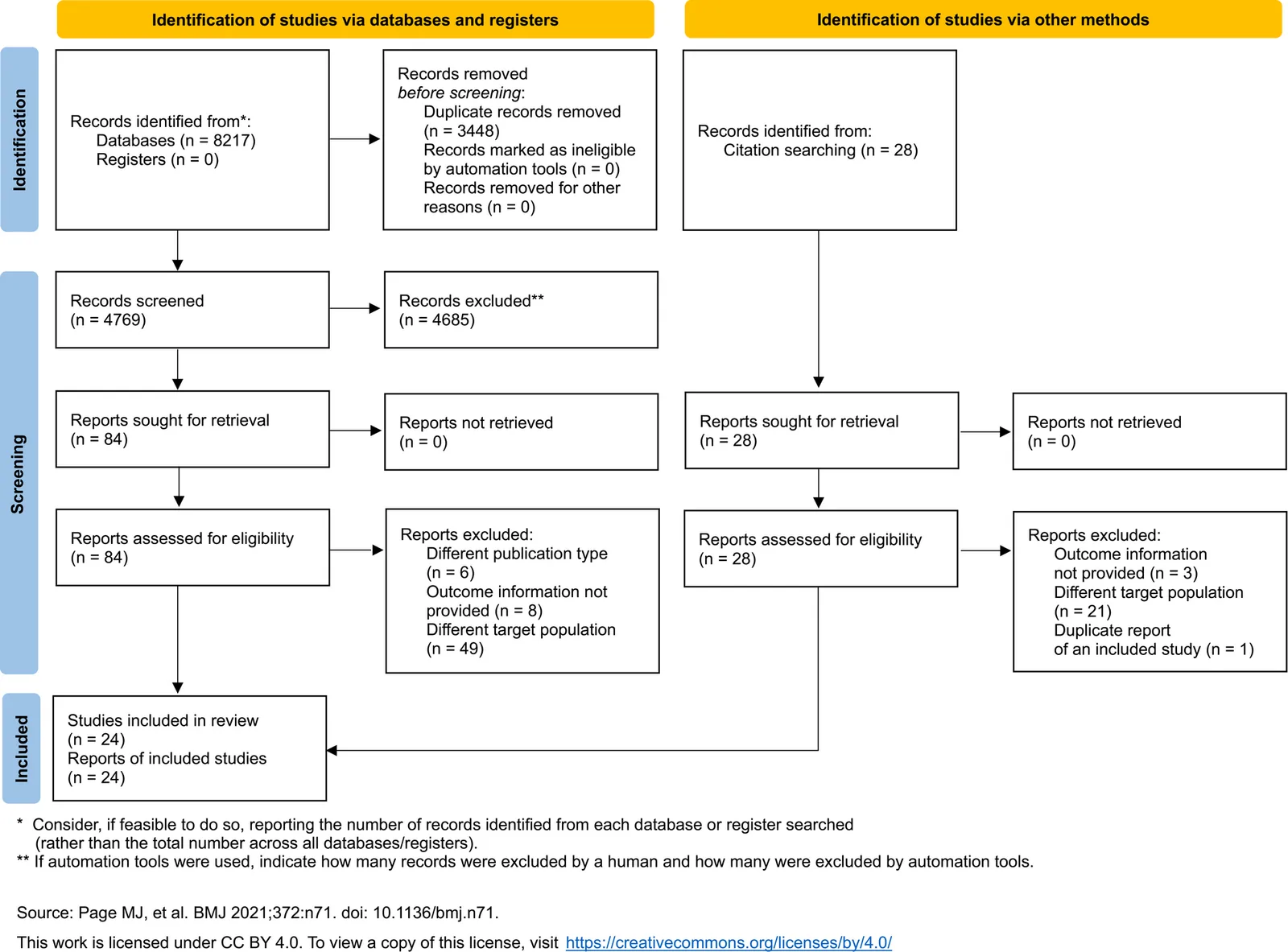
Search strategy flowchart based on the PRISMA 2020 statement. Note. An updated guideline for reporting systematic reviews [13]

Figure 1 depicts the study selection process. Twenty-four studies were included. After removing 4,493 duplicates, two reviewers independently screened titles and abstracts, excluding 4,685 records based on predefined criteria. Exclusions included seven publications without academic research design (e.g., case reports, discussion papers, commentaries, editorials, letters, expert opinions, news articles, conference abstracts, reviews without full text, and non-peer-reviewed publications), two studies with ineligible populations, 384 studies lacking outcome data, and 142 articles published in non-English languages. Subsequently, 61 full-text articles were assessed for eligibility, resulting in the inclusion of 21 studies from the database search. Backward citation searching of the reference lists of these included studies identified 28 additional records, 3 of which met the eligibility criteria, yielding a final total of 24 studies in this review.

### Overview

Of the 24 studies included in this scoping review, three were published between 1991 and 2009 [14–16], four between 2010 and 2014 [17–20], six between 2015 and 2019 [21–26], and 11 between 2020 and 2025 [27–37] (Additional file 2).

Most included studies were intervention studies, comprising ten randomized controlled trials [17–22, 29, 31–33, 37] and four quasi-experimental studies [14, 21, 27, 30]. These studies compared specific perioperative nursing practices by operating room nurses and examined intervention-related outcomes. Additionally, several observational studies were also included.

Geographically, the studies spanned various countries: six from Turkey, four from China, three from Sweden, two from Japan, and two from the United States. Single studies were conducted in Spain, Switzerland, Ireland, Finland, Australia, Iran, and Taiwan.

### Outcomes reported

**Fig. 2:**
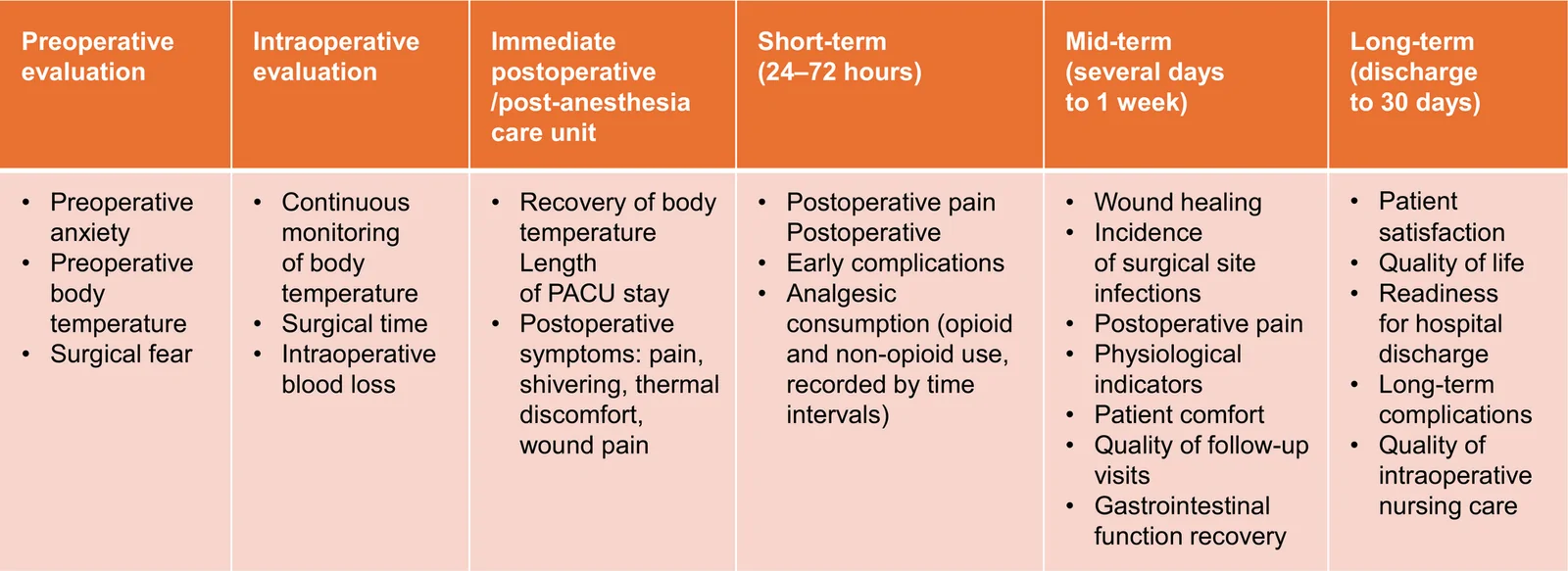
Perioperative outcomes associated with operating room nursing practice categorized by evaluation time points. Note. This figure summarizes outcomes of perioperative nursing interventions provided by operating room nurses, classified as preoperative, intraoperative, immediate postoperative, short-term, mid-term, and long-term evaluations

This scoping review maps the outcomes of perioperative nursing practices conducted by operating room nurses across 24 studies, using various outcome measures from the preoperative period to long-term recovery. The timing of these evaluations aligned with intervention phases, capturing immediate to long-term outcome patterns systematically (Additional file 3; Fig. 2).

Many studies assessed outcomes across multiple perioperative phases. Consequently, the total number of reported outcomes by phase exceeded the total number of included studies. The following sections describe the specific outcomes and their measurement methods, organized by each perioperative phase.

### Preoperative evaluation

Outcomes were assessed during the preoperative phase in 11 included studies. Psychological outcomes predominated, with preoperative anxiety being the most frequently measured outcome, reported in five studies [14, 17, 29–31]. Physiological baseline indicators, particularly preoperative temperature, were assessed in five studies [18–23, 32], while surgical fear was reported in one study [33]. Overall, preoperative outcomes primarily captured patients’ psychological readiness and baseline physiological status.

### Intraoperative evaluation

Seven studies evaluated intraoperative outcomes. The most frequently reported outcomes were continuous body temperature monitoring [18–23, 32], along with intraoperative blood loss, reported in three studies [22, 24, 34], and operative time, reported in two studies [24, 34]. These outcomes primarily reflected continuous physiological monitoring and objective indicators of surgical efficiency during the intraoperative phase.

Overall, intraoperative outcomes primarily captured thermal regulation, procedural efficiency, and objective surgical process indicators during active perioperative management.

### Immediate postoperative and post-anesthesia care unit evaluation

Five studies evaluated outcomes during the immediate postoperative and post-anesthesia care unit (PACU) phases. Recovery of body temperature was assessed in five studies [18–23, 32], while immediate postoperative symptoms were reported in two studies [22, 32]. One study additionally reported PACU length of stay [20]. These outcomes primarily reflected early postoperative physiological stabilization and symptom recovery.

Overall, outcomes in this phase primarily captured postoperative thermal recovery, early symptom burden, and short-term physiological stabilization.

### Short-term postoperative evaluation (24–72 h)

Seven studies assessed outcomes within 24–72 h after surgery. Pain-related outcomes were the most frequently reported, evaluated in seven studies [17, 19, 22, 29–31, 34], followed by anxiety in three [29–31], early postoperative complications in three [17, 22, 31], and medication use in two [19, 30]. These outcomes primarily reflected symptom burden, psychological status, early complications, and treatment needs during short-term postoperative recovery.

Overall, short-term postoperative outcomes primarily captured symptom control, early complication monitoring, and evolving recovery needs within the first 72 postoperative hours.

### Mid-term postoperative evaluation (several days to 1 week)

Nine studies evaluated outcomes from several days to 1 week after surgery. Wound healing and infection prevention were reported in four studies [26, 27, 35, 37], gastrointestinal function recovery in two studies [34, 37], postoperative comfort in one [36], continued pain-related outcomes in three [25, 31, 34], quality of postoperative follow-up in one [15], and physiological indicators in one [35]. These outcomes primarily reflected wound status, recovery progression, symptom burden, and postoperative physiological responses during the mid-term recovery phase.

Overall, mid-term postoperative outcomes primarily captured wound healing trajectories, recovery progression, ongoing symptom burden, and physiological recovery responses within the first postoperative week.

### Long-term postoperative evaluation (discharge to 30 days)

Eight studies evaluated long-term postoperative outcomes from discharge to 30 postoperative days. Patient satisfaction was the most frequently reported outcome, described in six studies [21, 26, 27, 31, 34, 35], followed by quality of life in two [27, 35], readiness for hospital discharge in one [28], and long-term complications in one [26]. Additionally, one study evaluated patients’ perceptions of the quality of intraoperative nursing care across multiple care dimensions at hospital discharge and approximately one month postoperatively [16]. These outcomes primarily reflected patients’ perceptions of care, recovery readiness, quality of life, and complication monitoring beyond the immediate postoperative period.

Overall, long-term postoperative outcomes primarily captured patient-reported recovery experiences, discharge preparedness, quality of life, and complication surveillance extending into the first postoperative month.

Collectively, the mapped outcomes progressed conceptually along the perioperative continuum, from preoperative psychological readiness, through intraoperative physiological stability and surgical-process indicators, to postoperative symptom control, early complications, and longer-term patient-centred outcomes. However, outcome definitions, measurement methods, and assessment time points varied considerably. For example, anxiety was assessed at different perioperative time points using the State–Trait Anxiety Inventory, body temperature was measured using different anatomical sites and monitoring methods, and postoperative pain was evaluated using different scales at varying postoperative intervals.

## Discussion

### Characteristics of the included studies

Most of the 24 studies in this scoping review were published after 2010, with a notable increase in number of studies published after 2020. Only three studies were published before 2010, suggesting that research evaluating outcomes associated with perioperative nursing practices performed by operating room nurses has gained increasing international attention in recent years. This trend may reflect the advancement of evidence-informed perioperative nursing, increased focus on patient-centered surgical care, and growing scholarly interest in clarifying nursing contributions across the perioperative continuum [38]. Many studies published after 2020 employed randomized controlled trial or quasi-experimental designs, comparing the presence or absence of operating room nurse-led care and examining associated postoperative outcomes. Consequently, aspects of nursing practice previously regarded as “tacit knowledge” may be increasingly described and systematized as explicit knowledge [39].

Considering geography, the largest number of studies were from Turkey (*n* = 6), followed by China (*n* = 4), Sweden (*n* = 3), Japan (*n* = 2), and the United States (*n* = 2). The pronounced research activity in these countries may reflect differences in nursing practice scope, institutional contexts, educational standards, and research priorities [38]. One study each was conducted in Spain, Switzerland, Ireland, Finland, Iran, Australia, and Taiwan, reflecting a growing international interest in outcomes related to operating room nursing practices. Patient experience and quality-of-life–related outcomes have increasingly been emphasized as important indicators in perioperative nursing research. Psychological and informational support from nurses has been evaluated in relation to these outcomes [27]. This aligns with the findings of this review, suggesting continued attention to the multifaceted role of operating room nurses beyond technical support, including contributions related to the patient experience.

Future challenges include identifying core elements of nursing interventions that may be applicable across cultural and institutional contexts, as well as standardizing outcome indicators. Furthermore, the studies reported that integrating such evidence into educational programs and institutional frameworks may support the clearer articulation and structuring of nursing expertise, ultimately contributing to safer and more meaningful perioperative experiences for patients.

### Characteristics and temporal patterns of outcomes

This scoping review reveals a diverse range of outcomes related to perioperative nursing practices by operating room nurses, covering the preoperative period through long-term postoperative recovery. Outcome evaluations closely aligned with the intervention phase and anticipated onset of outcome changes, creating a framework for assessing immediate -to long-term patterns. This temporal stratification corresponds to established perioperative outcome evaluation frameworks [40], suggesting that different types of interventions may be associated with distinct outcome patterns at various perioperative phases.

In the preoperative phase, psychological indicators were predominant, with anxiety and surgical fear as key outcomes. These factors have been reported in previous literature in relation to postoperative pain, recovery time, and patient satisfaction [41], emphasizing the need for preoperative psychological preparation. Intraoperative outcomes primarily involved physiological parameters, predominantly temperature management (four studies), suggesting that operating room nurses may contribute to supporting patient safety during this phase.

In the postoperative phase, eight studies evaluated long-term outcomes. These findings suggest that operating room nurses’ interventions may indicate possible contributions related not only to patients’ short-term recovery but also to their long-term quality of life, reintegration into daily activities, safety, and functional outcomes.

Overall, this study identifies outcomes varying by perioperative phase: psychological preparation in the preoperative phase, physiological stability and surgical efficiency during the operation, physiological stability during immediate postoperative recovery, and patient-centered outcomes such as satisfaction and quality of life in the long term. This shift from immediate safety indicators to long-term patient-reported outcomes suggests increasing attention toward comprehensive, patient-centered perioperative care [42].

### Methodological challenges and the need for standardization

Although similar conceptual domains were evaluated, measurement methods and timing significantly varied across studies. Standardized instruments, such as the STAI for anxiety and the visual analog scale for pain, were commonly used; however, the timing, frequency, and duration of follow-up assessments differed widely [43]. This heterogeneity poses a challenge in perioperative research [44], hindering evidence synthesis and meta-analysis.

To address this challenge, developing a core outcome set tailored to perioperative nursing may be valuable. A core outcome set represents an agreed minimum set of outcomes that should be measured and reported in all studies within a specific field [45]. Initiatives such as the Standardized Endpoints in Perioperative Medicine project [40] and the Pediatric Perioperative Outcomes Group [46] demonstrate the value of such frameworks. Based on this review, five potential key outcome domains can be considered for perioperative nursing: (1) preoperative psychological preparation, (2) intraoperative physiological stability, (3) immediate postoperative recovery, (4) short-term functional recovery, and (5) long-term patient-centered outcomes. Future research may benefit from consensus-building among patients, clinicians, and researchers to establish this core outcome set.

The use of patient-reported outcomes in long-term follow-up is limited. Beyond patient satisfaction, few studies assess broader indicators such as functional status, symptom burden, or quality of life. Patient-reported outcome measures capture recovery aspects not fully reflected by traditional clinical indicators, such as daily functioning and return to normal life [47]. Systematically integrating patient-reported outcome measures into future research may further support the evaluation of perioperative nursing interventions. The heterogeneity observed across studies likely reflects differences in clinical contexts, intervention complexity, and outcome prioritization, highlighting the need for greater consensus in outcome selection.

### Limitations

This scoping review has several limitations. First, the search was restricted to studies published in English, excluding non-English publications and certain types of gray literature (e.g., conference abstracts, non-peer-reviewed materials) owing to resource constraints. Consequently, some relevant studies may have been overlooked, resulting in selection bias. Second, the included studies exhibited heterogeneity in study populations, intervention content, outcome definitions, measurement methods, and evaluation time points, which, although common in scoping reviews, limited the feasibility of direct comparisons or integrated assessments and precluded firm conclusions regarding effectiveness. Third, because no formal critical appraisal of methodological quality was performed, consistent with scoping review methodology, the findings should be interpreted as a map of the outcomes reported in the literature rather than as evidence of the effectiveness of perioperative nursing practices. Despite these limitations, the review synthesized the available evidence and provided a foundation for future research planning and the selection of outcome measures.

## Conclusions

This scoping review maps that the outcomes of perioperative nursing practices by operating room nurses have been evaluated across various time points and outcome measures in existing research. Assessments included preoperative psychological status, intraoperative physiological stability, immediate postoperative recovery, short-term symptoms and complications, mid-term wound healing and patient comfort, and long-term quality of life and reintegration.

These findings provide insights for planning outcome evaluations in clinical practice and for selecting outcome measures in nursing education and research. Establishing a comprehensive evaluation framework incorporating long-term, patient-centered outcomes may support the scientific validation and clinical implementation of operating room nurses’ expertise. Future efforts may benefit from developing standardized core outcome sets and conducting longitudinal follow-up studies on quality of life and functional recovery, as well as employing mixed-method approaches to capture patient-centered outcomes.

## Supplementary Information

Below is the link to the electronic supplementary material.

Supplementary Material 1: Additional file 1: This file presents the detailed search terms and strategies used for each database

Supplementary Material 2: Additional file 2: The table presents the article title, author(s), reference number, country of publication, study purpose, study design, study methods, and key findings of the included studies

Supplementary Material 3: Additional file 3: This file presents the title, author(s), reference number, outcomes, perioperative nursing practices, study participants, outcome measurement methods, and outcome assessment time points

## Data Availability

No datasets were generated or analysed during the current study.

## Abbreviations

PACU: Post-anesthesia care unit
STAI: State-Trait Anxiety Inventory
