# Supplementary material for: Outcomes of perioperative nursing practices performed by operating room nurses: a scoping review
Source: BMC Nurs. 2026 Aug 3;25:1036. doi: 10.1186/s12912-026-05104-x (PMC13721582; doi:10.1186/s12912-026-05104-x)
Supplement: Supplementary file 1 [file 12912_2026_5104_MOESM1_ESM.docx]

**Additional file 1**: Search strategy

**Pubmed**

(“operating room nursing”[MeSH Terms] OR “operating room nursing”[Title/Abstract] OR “perioperative nursing”[MeSH Terms] OR “perioperative nursing”[Title/Abstract] OR “surgical nursing”[All Fields] OR “medical surgical nursing”[MeSH Terms] OR “medical surgical nursing”[Title/Abstract]) AND (“preoperative care”[MeSH Terms] OR “preoperative care”[Title/Abstract] OR “preoperative period”[MeSH Terms] OR “preoperative period”[Title/Abstract] OR “postoperative care”[MeSH Terms] OR “postoperative care”[Title/Abstract] OR “postoperative period”[MeSH Terms] OR “postoperative period”[Title/Abstract])

**Embase**

(exp “operating room nursing”/ OR “operating room nursing”.tw. OR exp “perioperative nursing”/ OR “perioperative nursing”.tw. OR “surgical nursing”.af. OR exp “medical surgical nursing”/ OR “medical surgical nursing”.tw.) AND (exp “preoperative care”/ OR “preoperative care”.tw. OR exp “preoperative period”/ OR “preoperative period”.tw. OR exp “postoperative care”/ OR “postoperative care”.tw. OR exp “postoperative period”/ OR “postoperative period”.tw.)

**CINAHL Plus**

((MH ”operating room nursing+”) OR (TI ”operating room nursing” OR AB ”operating room nursing”) OR (MH ”perioperative nursing+”) OR (TI ”perioperative nursing” OR AB ”perioperative nursing”) OR ”surgical nursing” OR (MH ”medical surgical nursing+”) OR (TI ”medical surgical nursing” OR AB ”medical surgical nursing”)) AND ((MH ”preoperative care+”) OR (TI ”preoperative care” OR AB ”preoperative care”) OR (MH ”preoperative period+”) OR (TI ”preoperative period” OR AB ”preoperative period”) OR (MH ”postoperative care+”) OR (TI ”postoperative care” OR AB ”postoperative care”) OR (MH ”postoperative period+”) OR (TI ”postoperative period” OR AB ”postoperative period”))
