# Supplementary material for: Outcomes of perioperative nursing practices performed by operating room nurses: a scoping review
Source: BMC Nurs. 2026 Aug 3;25:1036. doi: 10.1186/s12912-026-05104-x (PMC13721582; doi:10.1186/s12912-026-05104-x)
Supplement: Supplementary file 2 [file 12912_2026_5104_MOESM2_ESM.docx]

**Additional file 2:** Characteristics of included studies

| Reference No. | Article title | Author | Country of publication | Purpose of the study | Study design | Study methods | Key findings |
| --- | --- | --- | --- | --- | --- | --- | --- |
| 13 | Preoperation interviews: A nursing intervention to reduce patients’ anxiety | O’Sullivan and Richardson (1991) | Australia | To evaluate the impact of preoperative interviews on state anxiety in patients awaiting surgery. | Quasi-experimental study (with control group) | Sixty patients were conveniently sampled and divided into treatment (n = 30) and control (n = 30) groups. The treatment group received perioperative nurse-conducted interviews, while the control group did not. Anxiety was measured using the State-Trait Anxiety Inventory, and ANCOVA was used for between-group comparisons. | Patients who received preoperative interviews had significantly lower anxiety scores than those who did not (p < .05). |
| 14 | Preoperative stress: An operating room nurse intervention assessment.　10.1016/j.jopan.2015.08.011 | Gürsoy et al. (2016) | Turkey | To evaluate the effect of preoperative visits by OR nurses on patients’ stress levels. | Quasi-experimental study | A total of 179 surgical patients admitted to a public hospital were included. The intervention group (n = 86) received a 20-minute preoperative visit and standardized education by OR nurses, while the control group (n = 93) received usual care. Data were collected using a questionnaire, patient satisfaction scores, and the Burford Distress Thermometer. | Preoperative visits by OR nurses significantly reduced preoperative stress. Patients reported the visits were “effective for stress reduction.” |
| 15 | Effect of preoperative nursing visit on preoperative anxiety and postoperative complications in candidates for laparoscopic cholecystectomy: a randomized clinical trial.　10.1111/scs.12022 | Sadati et al. (2013) | Iran | To evaluate the effect of preoperative nurse visits on preoperative anxiety and postoperative complications in patients undergoing laparoscopic cholecystectomy. | Prospective, quasi-experimental, randomized clinical trial | A total of 100 laparoscopic cholecystectomy candidates were enrolled in an Iranian hospital (Feb 2010–Jan 2011). The intervention group received two preoperative interviews, while the control group received routine nursing. Anxiety was assessed using the Spielberger State-Trait Anxiety Inventory, and postoperative pain, nausea, vomiting, and other complications were recorded. Analyses included independent-samples t-tests using SPSS 16. | The intervention group reached Aldrete score 9 faster (18 vs 30 min), had lower incidence of postoperative nausea and vomiting (6% vs 20%, P=0.04), lower postoperative pain (Visual Analog Scale: 5.1 vs 5.7, P=0.001), and shorter time to ambulation (0.9 vs 1.2 days, P=0.002). Preoperative nursing visits also reduced preoperative anxiety. |
| 16 | A comparison of warming interventions on the temperatures of inpatients undergoing colorectal surgery. | Nicholson (2013) | United States | To compare the effect of preoperative warming using a forced-air warming system on body temperature upon arrival in the recovery room among hospitalized patients undergoing colorectal surgery. | Prospective, repeated-measures design, randomized controlled trial | Sixty-six patients were randomly assigned to two groups (control: cotton blanket, n = 32; intervention: forced-air warming gown, n = 34). The intervention consisted of at least 30 minutes of preoperative warming. Body temperature was measured and compared preoperatively, intraoperatively, and postoperatively. Student’s t-test and chi-square test were used for statistical analysis. | No significant difference in hypothermia incidence was observed between cotton blanket and forced-air warming groups during pre-, intra-, and postoperative periods (P = .05). Prewarming may contribute to normothermia immediately after surgery. |
| 17 | Efficacy of forced‐air warming for preventing perioperative hypothermia and related complications in patients undergoing laparoscopic surgery: A　randomized controlled trial.  10.1111/ijn.12660 | Su and Nieh (2018) | Taiwan | To examine whether forced-air warming therapy is effective in preventing perioperative hypothermia and reducing complications in patients undergoing laparoscopic surgery. | Randomized controlled trial | Between January and November 2015, 127 patients scheduled for laparoscopic thoracic or abdominal surgery at a 1,500-bed teaching hospital were randomized to a forced-air warming group (n = 64) or passive insulation group (n = 63). Outcomes included core and skin temperature, shivering, thermal discomfort, blood loss, and cardiac adverse events. Analyses were conducted using IBM SPSS Statistics 22.0 with Kolmogorov–Smirnov test, chi-square test, independent t-test, and generalized estimating equations. | Forced-air warming was more effective than passive insulation. Intraoperative blood loss was greater in the control group, suggesting hypothermia may increase bleeding. Findings support the role of OR nurses in maintaining normothermia to reduce blood loss and transfusion needs. |
| 18 | A nurse-led audit on the incidence and management of inadvertent hypothermia in an operating theatre department of an Irish hospital | Healy et al. (2018) | Ireland | To evaluate the incidence and management of unintended perioperative hypothermia in the OR and recovery room. | Quantitative observational study | Between November 2016 and February 2017, 100 patients (pediatric and adult) were included. Patient demographics, type of surgery, risk factors, perioperative temperatures (measured at five intervals with a tympanic thermometer), and warming interventions (warm blankets, forced-air warming, thermal caps, leggings, warm IV fluids) were recorded. Descriptive statistics were performed. | The incidence of unintended perioperative hypothermia was high, with inconsistent documentation and prevention practices. The forced-air warming device Bair Hugger™ was the most commonly used warming intervention. |
| 19 | Improvements in thoracic surgery outcomes: a multi-institutional collaboration study. 10.1186/s13019-015-0228-7 | Iwasaki et al. (2015) | Japan | To evaluate the impact of standardization of thoracic surgical tasks by thoracic surgeons and OR nurses across multiple institutions on surgical processes and patient outcomes. | Multicenter before-and-after comparative study | Thoracic surgical practices were standardized across three university-affiliated hospitals between 2011 and 2012. Surgical time and blood loss before and after standardization were compared using the Wilcoxon rank-sum test. | Standardized interventions significantly reduced surgical time and intraoperative blood loss after implementation. |
| 20 | The clinical effect of nursing intervention in the operating room on the prevention of orthopedic wound infections | Bai et al. (2021) | China | To examine the effect of OR nursing interventions on the prevention of wound infections in elderly patients undergoing orthopedic surgery. | Quasi-experimental study (comparison between intervention and control groups) | A total of 128 orthopedic patients were divided into observation (n = 64) and control groups (n = 64). Outcomes compared included grade A incision healing rate, time to oral intake and ambulation, length of stay, incidence of wound infection, prognosis, Quality of Life scores, and overall satisfaction. Interventions included preoperative visits and psychological support, enhanced intraoperative management, aseptic technique, warming care, and postoperative wound care. | The observation group showed significantly better wound healing (Grade A), higher quality of life, lower surgical site infection rates, and higher satisfaction. Postoperative SSI incidence was 0% in the observation group versus 21.88% in controls. OR nursing interventions effectively prevented SSI and promoted wound healing. |
| 21 | Cardiac surgery patients' evaluation of the quality of theatre nurse postoperative follow-up visit. DOI 10.1016/j.ejcnurse.2008.07.005 | Falk-Brynhildsen and Nilsson (2009) | Sweden | To evaluate the quality of postoperative follow-up visits conducted by OR nurses and to clarify whether this quality is associated with patients’ sex and type of admission (elective or emergency). | Prospective exploratory study | Data were collected from　78　cardiac surgery patients who received postoperative follow-up visits, using a modified 16-item “Quality from the Patient’s Perspective” questionnaire between October and December 2005. Participants completed the survey anonymously at the hospital or at home. Descriptive statistics were presented as means and standard deviations. Group differences were analyzed using the Mann–Whitney U test (p < 0.05). A “Perceived Quality Index” score was calculated based on perceived reality and subjective importance. | Patients evaluated follow-up visits positively overall. Emergency surgery patients rated six items, including reduction of postoperative anxiety, understanding of surgical content, and explanation of physical condition, significantly higher than elective surgery patients. Female patients rated “the nurse listened to me carefully” and “the nurse understood my feelings” higher than male patients. |
| 22 | Examining the relationship between perceived readiness for hospital discharge and quality of perioperative nursing care in ambulatory surgery patients.　10.4274/BMJ.galenos.2024.2024.4-2 | Güven et al. (2024) | Turkey | To assess the relationship between perceptions of discharge readiness and the quality of perioperative nursing care in patients undergoing ambulatory surgery. | Descriptive, correlational design | Between October 2023 and March 2024, 124 outpatients undergoing surgery at a training hospital in Istanbul were assessed. Data were collected pre-discharge using the Good Perioperative Nursing Care Scale and the Readiness for Hospital Discharge Scale. Analyses included descriptive statistics, Kolmogorov–Smirnov test, t-test, and Mann–Whitney U test. | 50.8% of patients were judged as not ready for discharge. Readiness was significantly associated with adequacy of discharge education. Mean scores of the Good Perioperative Nursing Care Scale and Readiness for Hospital Discharge Scale showed a significant moderate positive correlation (r = 0.633, p < 0.001). The quality of perioperative nursing care influenced discharge readiness. |
| 23 | The effect of preoperative nursing visit on anxiety and pain level of patients after surgery.　10.1016/j.jopan.2022.05.086 | Aydal et al. (2023) | Turkey | To evaluate the impact of preoperative visits by OR nurses on preoperative anxiety and postoperative pain levels in patients undergoing laparoscopic surgery. | Randomized controlled trial | Data were collected from 135 patients undergoing laparoscopic surgery in Istanbul between March and October 2019. Seventy-two patients received an operating room nurse visit, and 63 received usual care by ward nurses. Anxiety and pain were measured using the State-Trait Anxiety Inventory and the Visual Analog Scale. | Preoperative anxiety was significantly reduced in the OR nurse visit group. However, no significant between-group differences were found for postoperative anxiety or pain. Preoperative nursing visits may serve as an effective method to reduce patient anxiety levels. |
| 24 | The effect of visits by operating room nurses before cardiac surgery on anxiety and pain management.　10.1016/j.jopan.2023.01.022 | Arpag et al. (2023) | Turkey | To evaluate the effect of preoperative visits by OR nurses on postoperative anxiety, frequency and intensity of pain, and analgesic use in patients undergoing cardiac surgery. | Quasi-experimental study (non-randomized, controlled pretest–posttest design) | Between August 2020 and April 2021, 64 patients undergoing coronary bypass surgery at a Turkish university hospital (32 intervention, 32 control) were studied. Anxiety and pain were measured using the State-Trait Anxiety Inventory and Visual Analog Scale. Analyses included both parametric and non-parametric tests using IBM SPSS Statistics 25.0. | The intervention effectively reduced postoperative state anxiety levels (P < .05). Although no significant difference was found in pain severity, the intervention reduced pain frequency (P < .05) and decreased opioid and non-opioid analgesic use during the first 12 hours (P < .05). Preoperative visits by OR nurses may reduce postoperative anxiety, pain frequency, and analgesic use. |
| 25 | Impact of an operating room nurse preoperative dialogue on anxiety, satisfaction, and early postoperative outcomes in patients undergoing major surgery | Dias et al. (2022) | Switzerland | To clarify the effects of preoperative dialogue conducted by OR nurses on patients’ anxiety, satisfaction, and postoperative outcomes. | Randomized controlled trial | Seventy patients undergoing elective major visceral surgery were included. Outcomes assessed were anxiety, satisfaction, postoperative pain, postoperative nausea and vomiting, opioid use, and length of stay. The intervention group received a 10-minute standardized interview delivered by specially trained OR nurses using a patient-centered approach. Analyses included Mann–Whitney U tests or Student’s t-tests for continuous variables and Fisher’s exact test for categorical variables. | Patient-centered preoperative dialogue was effective in reducing preoperative anxiety among patients undergoing major surgery. |
| 26 | Effects of postoperative active warming and early exercise on postoperative body temperature distribution: Non-blinded and randomized controlled trial.　10.1111/jjns.12335. | Kameda et al. (2020) | Japan | To examine the effectiveness of postoperative active warming and mobilization on body temperature and peripheral circulation in patients undergoing major abdominal surgery. | Prospective, non-blinded, randomized controlled trial | Fifty-one patients were allocated to a control group, an exercise group, or a warming group. Core temperature, skin temperature, and circulatory indices were continuously monitored from anesthesia induction until 12 hours after ward arrival, and group comparisons were conducted. | In the warming group, skin temperature rose rapidly within 1 hour postoperatively and remained higher than in control and exercise groups. Early mobilization significantly improved peripheral circulation (perfusion index). |
| 27 | The effect of preoperative education regarding intraoperative care on a patient's level of fear of surgery: A randomized controlled study.　10.1002/aorn.14129 | Özkan and Yıldızelı Topçu (2024) | Turkey | To investigate the impact of preoperative education regarding the surgical environment and procedures on fear levels related to surgery in patients undergoing abdominal surgery. | Randomized controlled trial | Ninety-two patients undergoing elective abdominal surgery at a university hospital in northwestern Turkey (October 2018–July 2019) were included. The intervention group (n = 46) received education about the OR environment and intraoperative care, while the routine care group (n = 46) received standard care. Surgical fear was measured using the Surgical Fear Questionnaire. Data were analyzed with SPSS 22.0 using t-tests and chi-square tests. | Patients receiving information about perioperative care and surgical processes had significantly lower fear scores upon OR arrival (P < 0.001). Educational content (explanation of OR environment and procedures) was effective in reducing surgical fear. |
| 28 | Effect of refined management in operating room nursing on surgical efficiency and nursing satisfaction during laparoscopic radical resection of colon cancer | Pan and Yi (2024) | China | To evaluate the impact of refined management in OR nursing during laparoscopic radical surgery for colon cancer on surgical efficiency and nursing satisfaction. | Retrospective comparative study | One hundred patients undergoing laparoscopic radical colectomy for colon cancer (Jan 2021–Jun 2023) were included. The observation group (n = 51) received refined management, while the control group (n = 49) received routine nursing. Surgical efficiency, care quality evaluation, pain scores (the Numerical Rating Scale), and nursing satisfaction were compared. Multivariate regression analysis was conducted. | The observation group had significantly shorter preoperative preparation time, operative time, intraoperative blood loss, and time to first postoperative defecation compared with controls (all P < .05). Refined management was an independent factor associated with improved prognosis. |
| 29 | Standardized operating room nursing care is effective in preventing the occurrence of surgical wound infections | Pei and Song (2025) | China | To investigate the effect of standardized nursing care in the OR on the incidence of surgical site infections. | Retrospective study | One hundred twenty-eight orthopedic surgical patients (64 control, 64 observation) were included. The control group received routine operating room nursing, while the observation group received standardized operating room nursing. Independent-samples t-tests were used for between-group comparisons, paired t-tests for within-group pre–post comparisons, and chi-square tests for categorical data. | Standardized OR nursing care was effective in preventing surgical site infections and contributed to healthier lifestyles and improved clinical outcomes. |
| 30 | The relationship between perioperative care quality and postoperative comfort level in patients with hip replacement surgery.　10.1016/j.jopan.2022.05.068 | İbrahimoğlu et al. (2023) | Turkey | To examine the relationship between the quality of perioperative nursing care and postoperative comfort in patients undergoing hip replacement surgery. | Descriptive study | Ninety hip replacement patients in a Turkish training hospital (February–September 2021) were evaluated using the Good Perioperative Nursing Care Scale and PHRCS. Patients were interviewed face-to-face on postoperative day 2, and perioperative data were extracted from OR records. Data were analyzed using Kolmogorov–Smirnov test, Mann–Whitney U, Kruskal–Wallis, and Spearman correlation tests. | Higher quality perioperative care was significantly associated with higher postoperative comfort (r = 0.253, P < .05). |
| 31 | Perioperative dialogue on postoperative recovery measured by the use of pain medication, psychopharmaceutical agents and length of hospital stay.　10.1177/2057158518754785 | Swenne et al. (2018) | Sweden | To quantitatively evaluate the effect of perioperative dialogue during surgery on postoperative analgesic consumption and length of hospital stay in cancer patients with severe peritoneal carcinomatosis. | Retrospective comparative study | Electronic medical records of 89 patients who underwent CRS and HIPEC at a university hospital were reviewed. Thirty-seven patients received perioperative dialogue and 52 did not. Data collected included demographics, disease-related variables, use of analgesics and psychotropics, and pain assessment scores. Statistical analysis was performed using IBM SPSS Statistics 20.0 with descriptive statistics, chi-square test, and Mann–Whitney U test. | Perioperative dialogue did not significantly affect analgesic or psychotropic use or length of stay. However, perioperative dialogue provided patient-centered information and support, potentially benefiting recovery. |
| 33 | Safety and satisfaction of patients with nurse's care in the perioperative.　10.1590/1518-8345.2646.3142 | Sillero‑Sillero and Zabalegui (2019) | Spain | To investigate the association between nursing care during the perioperative period and patients’ safety and satisfaction. | Cross-sectional, multilevel, correlational study | Data were collected from 105 surgical nurses and 150 surgical patients in a tertiary hospital. Nurse variables included demographics, work environment perception, burnout, satisfaction, and turnover intention. Patient variables included demographics, type of surgery, comorbidities, length of stay, and adverse events. Analyses included multistage analysis, chi-square test, and Fisher’s exact test. | Surgical nursing specialization was significantly associated with adverse event rates. Nurse characteristics (sex, education, role, and contract status) were not associated with overall patient satisfaction. |
| 34 | The perioperative dialogue reduces postoperative stress in children undergoing day surgery as confirmed by salivary cortisol. 10.1111/j.1460-9592.2011.03656.x | Wennström et al. (2011) | Sweden | To evaluate the effect of perioperative dialogue on postoperative stress in children aged 5 to 11 years undergoing day surgery, using salivary cortisol as an indicator. | Randomized controlled trial | Ninety-three patients were randomly assigned to a standard care group, a preoperative information group, and a perioperative dialogue group (31 each). Saliva samples were collected pre- and post-operatively for cortisol measurement and analysis. | Children receiving continuous care through perioperative dialogue had significantly lower postoperative salivary cortisol levels (P = 0.003). Morphine consumption also differed between groups, suggesting perioperative dialogue reduces postoperative stress. |
| 35 | The quality of intraoperative nursing care: the patient’s perspective | Leinonen et al. (1996) | Finland | To document patients’ responses to intraoperative care, to examine whether their opinions changed over time, and to develop a patient-based scale for evaluating the quality of intraoperative nursing care. | Observational study (descriptive, cross-sectional; patient-perspective survey at two time points) | Structured questionnaire covering seven quality dimensions (biological–physiological, experiential, cognitive, functional, ethical, characteristics/competence of nursing professionals, and environmental), administered at the end of the hospital stay (n = 246) and about one month later at home (n = 158); measured with visual analogue scales and structured multiple-choice items. | Patients were generally satisfied with intraoperative nursing care. The most positive evaluations concerned the biological–physiological dimension, whereas the cognitive and experiential dimensions received the lowest ratings. The study highlighted the need to keep patients warm in the operating theatre and to attend to emergency, younger, and short-stay patients. |
| 36 | Effect of preoperative forced-air warming on postoperative temperature and postanesthesia care unit length of stay | Fettes et al. (2013) | United States | To evaluate whether preoperative forced-air warming reduces the number of patients arriving hypothermic in the PACU and shortens PACU length of stay. | Randomized controlled trial | 128 surgical patients randomly assigned to preoperative forced-air warming (n = 54) or no preoperative warming (n = 74). Core temperature was measured with a temporal artery-scanning thermometer preoperatively, on exiting the preoperative area, and on PACU admission; PACU length of stay was recorded. | Preoperative warming had no significant effect on postoperative temperature or PACU length of stay. PACU-admission temperature was 98.0°F (control) versus 98.1°F (warming) (P = .314), and median PACU stay was 50 versus 49 minutes (P = .545). No adverse temperature events occurred. |
| 37 | Application of operating room nursing intervention to incision infection of patients undergoing gastrointestinal surgery can reduce complications and improve gastrointestinal function | Wang et al. (2022) | China | To observe the influence of operating room nursing intervention on incision infection and gastrointestinal function recovery in patients undergoing gastrointestinal surgery. | Randomized controlled trial | 340 patients undergoing gastrointestinal surgery allocated by random number table to an operating room nursing group (n = 170) or a conventional nursing group (n = 170). Outcomes included incision infection signs, wound healing grade (A/B/C), gastrointestinal function indicators (anal exhaust, first defecation, gastric tube removal times), postoperative complications, and nursing satisfaction. | The operating room nursing group showed significantly lower incision swelling, pain, and secretion; higher grade A and lower grade B/C healing; shorter times to anal exhaust, first defecation, and gastric tube removal; fewer postoperative complications; and higher total satisfaction (93.53% vs 75.29%, χ² = 30.637, P < 0.001). All comparisons P < 0.05. |

Note: OR, operating room
