# Supplementary material for: Outcomes of perioperative nursing practices performed by operating room nurses: a scoping review
Source: BMC Nurs. 2026 Aug 3;25:1036. doi: 10.1186/s12912-026-05104-x (PMC13721582; doi:10.1186/s12912-026-05104-x)
Supplement: Supplementary file 3 [file 12912_2026_5104_MOESM3_ESM.docx]

**Additional file 3:** Characteristics of outcomes

| Reference No. | **Title** | **Author** | **Outcomes** | **Perioperative nursing practices** | **Study participants** | **Outcome measurement methods** | **Outcome assessment time points** |
| --- | --- | --- | --- | --- | --- | --- | --- |
| **13** | Preoperation interviews: A nursing intervention to reduce patients’ anxiety | O’Sullivan and Richardson | Patient anxiety | Preoperative interviews focusing on reducing patients’ fear of the unknown related to surgery. | 60 English-speaking adults (aged 18–65 years) undergoing scheduled surgery | State-Trait Anxiety Inventory score | After preoperative interview, on the evening before surgery |
| **14** | Preoperative stress: An operating room nurse intervention assessment.　10.1016/j.jopan.2015.08.011 | Gürsoy et al. | Preoperative stress and postoperative stress Patient satisfaction | Preoperative visit by OR nurses on the day before surgery to provide individualized support | 179 general surgical inpatients | Patient satisfaction score  Burford Distress Thermometer | Preoperative period   Postoperative period (not specified) |
| **15** | Effect of preoperative nursing visit on preoperative anxiety and postoperative complications in candidates for laparoscopic cholecystectomy: a randomized clinical trial.　10.1111/scs.12022 | Sadati et al. | Preoperative anxiety Postoperative complications (pain, nausea, recovery of consciousness, time to ambulation) | Preoperative day nurse visit providing individualized consultation and education. | 100 female patients scheduled for laparoscopic cholecystectomy (50 intervention, 50 control) | Spielberger State–Trait Anxiety Inventory, Postoperative Visual Analog Scale (pain), Time to vital sign stabilization, Incidence of nausea/vomiting, Aldrete score: time to reach recovery criteria, Time to first mobilization (from ward records) | On admission to surgical ward  Immediately before OR entry  24 hours postoperatively (pain, nausea, and vomiting) |
| **16** | A comparison of warming interventions on the temperatures of inpatients undergoing colorectal surgery. | Nicholson | Body temperature at post-anesthesia care unit arrival | Preoperative warming for 30 minutes; using forced-air warming versus conventional blanket insulation. | 66 adult inpatients (aged ≥18 years) scheduled for colorectal surgery | Pre- and postoperative temperature: portable electronic oral thermistor Intraoperative temperature: esophageal/rectal probe or temperature-sensing urinary catheter (selected by anesthesiologist) | Preoperative: after 30 min warming session  Intraoperative: first body temperature record within 15 min after induction  Postoperative: endpoint set at 15 min after post-anesthesia care unit arrival |
| **17** | Efficacy of forced‐air warming for preventing perioperative hypothermia and related complications in patients undergoing laparoscopic surgery: A　10.1111/ijn.12660  randomized controlled trial. | Su and Nieh | Presence of perioperative hypothermia;  Reduction of postoperative complications ; Blood loss ; Pain intensity;  Shivering level | Intervention group: intraoperative and post-anesthesia care unit warming with a forced-air warming device (3M Bair Hugger 775) set at 43°C. Control group: intraoperative passive insulation with heated blankets; in the post-anesthesia care unit, radiant warming system (RWS, 100 W/cm²) was used. | 127 patients undergoing abdominal laparoscopic surgery (64 intervention, 63 control) | Intraoperative esophageal temperature monitoring,  Preoperative and post-anesthesia care unit, tympanic temperature measured every 30 minutes,  Intraoperative blood loss (mL) from surgical records ,  Time until post-anesthesia care unit arrival, temperature recovered to 36°C (minutes),  Pain scores using VAS , Presence and severity of shivering a,  ECG and clinical records checked for events prior to post-anesthesia care unit transfer | Preoperative: in the OR waiting room  Intraoperative: esophageal temperature measured every 30 minutes by anesthesiologists (until the end of surgery); post-anesthesia care unit: tympanic temperature measured every 30 minutes; time to rewarming until 36°C recorded  ; Postoperative: pain, shivering, and blood loss evaluated before post-anesthesia care unit transfer;   Cardiac complications: assessed intraoperatively and postoperatively as needed |
| **18** | A nurse-led audit on the incidence and management of inadvertent hypothermia in an operating theatre department of an Irish hospital | Healy et al. | Incidence of unintended perioperative hypothermia | Thermal interventions: use of warmed blankets, forced-air warming device (Bair Hugger™), warming caps and leggings, and warmed IV fluids. Standard care was provided based on regional policies for the prevention and management of inadvertent perioperative hypothermia, including temperature monitoring and proactive rewarming interventions. | 100 surgical patients (13 pediatric and 87 adult; 25% elective, 72% emergency) | Anesthesia and nursing records (electronic or paper) for temperature measurements and documentation; Documentation of warming interventions (warmed blankets, forced-air warming device, warming cap, leggings, warmed IV fluids) | (1) OR reception arrival (2) OR entry (3) OR exit (4) Post-anesthesia care unit arrival (5) Post-anesthesia care unit discharge At each stage, body temperature measured and warming interventions recorded |
| **19** | Improvements in thoracic surgery outcomes: a multi-institutional collaboration study. 10.1186/s13019-015-0228-7 | Iwasaki et al. | Operation time blood loss (intraoperative blood loss) | Standardization of surgical tasks (e.g., preparation checklist, introduction of carts into the OR, arrangement of instruments, patient verification, time-out procedure, positioning and fixation, disinfection, temperature management) | All thoracic surgery cases at five medical university hospitals | Operative records: operative time (from incision to closure)  Intraoperative blood loss (mL) extracted from operative records | One month before and after standardization |
| **20** | The clinical effect of nursing intervention in the operating room on the prevention of orthopedic wound infections | Bai et al. | Degree of postoperative wound healing Incidence of postoperative wound infection Patient quality of life nursing satisfaction | Standardized nursing care: temperature and humidity control, aseptic techniques, equipment management, staff allocation, and infection prevention education. | 128 elderly patients undergoing orthopedic surgery (64 observation, 64 control) | Clinical records: wound healing, nutrition/feeding, mobilization, length of stay, wound infection rate, Quality of life questionnaires, Self-developed patient satisfaction questionnaires | During hospitalization and at discharge |
| **21** | Cardiac surgery patients' evaluation of the quality of theatre nurse postoperative follow-up visit. DOI 10.1016/j.ejcnurse.2008.07.005 | Falk-Brynhildsen and Nilsson | Quality of follow-up visits as evaluated by patients | Standardized information provision (e.g., explanation of postoperative course until discharge) and individualized conversation (reflection and future outlook) | 78postoperative cardiac surgery patients | Patient assessment scale (modified 16-item version of “Perceived Reality” and “Subjective Importance”) | Postoperative days 2–6 (average number of days 4) |
| **22** | Examining the relationship between perceived readiness for hospital discharge and quality of perioperative nursing care in ambulatory surgery patients.　10.4274/BMJ.galenos.2024.2024.4-2 | Güven et al. | Quality of nursing care Readiness for hospital discharge score | Perioperative nursing care for day-surgery patients, structured according to seven subdimensions: physical care, information provision, support, respect, personal characteristics, environment, and nursing process. | 124 outpatients undergoing surgery (mean age 43.1 years, 56.5% male) | Good Perioperative Nursing Care Scale  Readiness for Hospital Discharge Scale | After outpatient surgery, before patient discharge |
| **23** | The Effect of Preoperative Nursing Visit on Anxiety and Pain Level of Patients After Surgery.　10.1016/j.jopan.2022.05.086 | Aydal et al. | Preoperative anxiety Postoperative pain | Preoperative visits and individualized education (20–30 minutes) by OR nurses, providing necessary information and education regarding surgical processes and nursing care practices. | 135 patients undergoing laparoscopic surgery (72 in the OR nurse group, 63 in the control group) | State-Trait Anxiety Inventory score and Visual Analog Scale for pain assessment | Anxiety: before education, after education, 24 hours postoperatively  Pain: 24 hours postoperatively |
| **24** | The effect of visits by operating room nurses before cardiac surgery on anxiety and pain management.　10.1016/j.jopan.2023.01.022 | Arpag and Öztekin | Postoperative anxiety Frequency and intensity of postoperative pain Analgesic use | Preoperative patient visit by OR nurses (conducted at 18:30 on the day before surgery). Provision of an information booklet (covering morning preparations, unit discharge, transfer to OR, anesthesia administration, ICU postoperative awakening, and pain assessment). Implementation of a patient visit protocol (addressing concerns, oral explanation of booklet contents, greeting in the OR, informing of nurse participation in surgery, guidance to physician for medical questions). Review and question and answers with the patient for about 10–15 minutes. On the day of surgery, welcoming the patient at the OR entrance and accompanying them. | Adult cardiac surgery patients (aged 18–75 years) | State-Trait Anxiety Inventory Pain assessment (Visual Analog Scale) Analgesic administration records | Preoperative: at least 12 hours before surgical intervention, state and trait anxiety levels measured  10–20 minutes post-interview  Pain monitoring: scheduled intervals Z0: 0–12 h (hourly) Z1: 13–24 h (hourly) Z2: 25–36 h (every 4 h) Z3: 37–48 h (every 4 h) Z4: 49–60 h (every 4 h) Z5: 61–72 h (every 4 h) Analgesic administration monitoring: A0: 0–12 h, A1: 13–24 h, A2: 25–36 h, A3: 37–48 h, A4: 49–60 h, A5: 61–72 h |
| **25** | Impact of an operating room nurse preoperative dialogue on anxiety, satisfaction, and early postoperative outcomes in patients undergoing major surgery | Dias et al. | Patient anxiety Patient satisfaction Postoperative pain Postoperative course | Standardized 10-minute patient-centered preoperative interview. | 70 consecutive patients undergoing elective major visceral surgery (35 intervention: received semi-structured preoperative nurse dialogue, 35 control: no nursing intervention) | State-Trait Anxiety Inventory Visual Analog Scale EORTC In-Patsat32 patient-reported questionnaire (anxiety, satisfaction) Nursing records of postoperative course, opioid consumption, length of stay) | Anxiety: measured preoperatively and postoperatively with the State-Trait Anxiety Inventory; postoperative anxiety assessed on POD 2 Patient satisfaction: at discharge Postoperative pain: evaluated with Visual Analog Scale up to POD 4 |
| **26** | Effects of postoperative active warming and early exercise on postoperative body temperature distribution: Non-blinded and randomized controlled　10.1111/jjns.12335 trial. | Kameda et al. | Core-to-skin temperature difference (temperature gap) Perfusion index Severity of shivering Thermal discomfort Wound pain | Control group: routine clinical care. Exercise group: early intermittent bedside mobilization (started immediately after ward admission, repeated every hour until 9 pm). Warming group: active warming with a forced-air warming blanket set at 38°C. | 51 postoperative hepatobiliary and pancreatic surgery patients (male, aged 50–80 years, BMI 18.5–25 kg/m²; 17 patients per group) | Core temperature (SpotOn™) Skin temperature (measured at chest, arm, thigh, lower limb with infrared thermometer; average skin temperature calculated using Ramanathan method) Perfusion index (continuous monitoring) Thermal discomfort Wound pain: 100-point Visual Analog Scale | Temperature: before anesthesia induction, during surgery, at the end of surgery, at extubation, on OR exit, at ward arrival, 15/30/45 minutes after ward arrival, then hourly up to 12 hours  Shivering: 12 hours after ward arrival  Thermal discomfort and wound pain: postoperative day 1 |
| **27** | The effect of preoperative education regarding intraoperative care on a patient's level of fear of surgery: A randomized controlled study.　10.1002/aorn.14129 | Özkan and Yıldızelı Topçu | Surgical fear level | Preoperative education using an “Information Form for the Perioperative Period,” covering OR environment, surgical process, intraoperative care, surgical instruments, surgical team members, questions at OR arrival, patient positioning, and interventions in the postoperative recovery ward. A 20–30 minute interview and education session was conducted in the patient’s room. | 92 patients undergoing elective abdominal surgery | Surgical Fear Questionnaire | Before education, and at OR arrival after education (3 time points) |
| **28** | Effect of refined management in operating room nursing on surgical efficiency and nursing satisfaction during laparoscopic radical resection of colon cancer | Pan and Yi | Surgical efficiency (preoperative preparation time, operation time, intraoperative blood loss, time to first defecation) Evaluation of quality of nursing care Pain score Nursing satisfaction | Refined management practices: preoperative preparation and inspection of surgical instruments, strengthened intraoperative communication and coordination, prompt postoperative response, and strict hygiene management. | 100 patients undergoing laparoscopic radical resection of colon cancer / laparoscopic radical colectomy for colon cancer (51 intervention, 49 control) | Pain: Numerical Rating Scale (NRS, 0–10 scale) Nursing satisfaction: self-developed questionnaire based on Ministry of Health tertiary hospital standards (50 points maximum) Surgical efficiency: preoperative preparation time, operation time, intraoperative blood loss, postoperative time to first defecation | Pain scores: at 12 h, 24 h, and 48 h postoperatively  Nursing satisfaction: postoperative period |
| **29** | Standardized operating room nursing care is effective in preventing the occurrence of surgical wound infections | Pei and Song | Wound healing  Incidence of wound infection  Quality of life  Patient satisfaction  Inflammatory response indicators | Standardized OR nursing care including: (1) standardized skill training for OR nurses, (2) standardized aseptic surgery, (3) preoperative care (patient assessment and individualized care planning), (4) intraoperative care (direct interaction with the patient), (5) postoperative care (individualized balanced nutrition, sleep management, and psychological support). | 128 patients requiring orthopedic surgery (64 control, 64 observation) | Postoperative wound healing (Grade A, B, C)  Incidence of infectious complications (number of SSIs/total cases)  Inflammatory response markers (WBC, CRP, IL-6)  Patient QOL at discharge (SF-36)  Satisfaction with nursing care (self-developed questionnaire) | Within 1 week postoperatively (observation period) and at discharge |
| **30** | The relationship between perioperative care quality and postoperative comfort level in patients with hip replacement surgery.　10.1016/j.jopan.2022.05.068 | İbrahimoğlu et al. | Postoperative comfort | Nursing care spanning the perioperative period (preoperative, intraoperative, and postoperative). | 90 hip replacement patients | Good Perioperative Nursing Care Scale／Post Hip Replacement Comfort Scale | Postoperative day 2 |
| **31** | Perioperative dialogue on postoperative recovery measured by the use of pain medication, psychopharmaceutical agents and length of hospital stay.　10.1177/2057158518754785 | Swenne et al. | Analgesic consumption and administration method Use of psychotropic medications (benzodiazepines) Length of hospital stay (days) Number of postoperative pain assessments with Numerical Rating Scale > 3 during 7 days Use of psychosocial support services | Perioperative dialogue: conversations with the same OR nurse or nurse anesthetist before, during, and after surgery | 37 patients undergoing cytoreductive surgery + HIPEC (intervention) vs 52 patients (control) | Pain: Numeric Rating Scale (Numerical Rating Scale, 0–10 scale) Anxiety/worry: patient self-reported descriptive data documented in records Length of hospital stay: number of days recorded by surgical ward nurses in the electronic medial record and nursing notes Medication use: all drug names, dosages, administration methods, days, and frequency recorded Psychosocial support: presence or absence of contact with social workers or hospital chaplains | Postoperative 7 days: number of Numerical Rating Scale > 3 pain assessments  Entire hospitalization: total use of analgesics and psychotropic drugs  Recovery period: use of psychosocial support services  Anxiety/worry: patient self-reports continuously documented during hospitalization |
| **33** | Safety and satisfaction of patients with nurse's care in the perioperative.　10.1590/1518-8345.2646.3142 | Sillero‑Sillero and Zabalegui | Incidence of adverse events (pressure injury, hospital-acquired infection, phlebitis, drug-related adverse events, postoperative complications, pain) Patient satisfaction | Preoperative supportive care Postoperative care (contributing to reduction of adverse events) Nursing interventions covering the entire perioperative period (pre-, intra-, and postoperative) Safety management practices based on the SENECA100 model | 150 surgical patients | Patient questionnaires and hospital records on adverse events (pressure injuries, hospital-acquired infections, phlebitis, drug-related AEs, postoperative complications, pain, etc.)  Independent patient satisfaction surveys conducted by hospitals | From admission until 30 days post-intervention / entire perioperative period (unspecified) |
| **34** | The perioperative dialogue reduces postoperative stress in children undergoing day surgery as confirmed by salivary cortisol. 10.1111/j.1460-9592.2011.03656.x | Wennström et al. | Postoperative stress Postoperative pain | Perioperative dialogue: continuous dialogue and care provided by the same nurse anesthetist throughout the preoperative, intraoperative, and postoperative periods; listening to children’s questions, thoughts, language, needs, expectations, and experiences, enhancing their sense of control, confidence, and trust through interpersonal interaction | 93 children aged 5–11 years (day surgery) | Salivary cortisol concentration (analyzed by ELISA) Wong-Baker (FACES) Pain Rating Scale (0–5 points; 0 = no pain, 5 = worst damage) Morphine consumption per unit (based on administration records) Length of stay in the post-anesthesia care unit | Saliva collection: post-communication　(before anesthesia induction, postoperative recovery) Pain assessment: three evaluations in the post-anesthesia care unit (periodically when child cooperation was indicated) Morphine use: recorded at each administration, total dose until recovery phase |
| **35** | The quality of intraoperative nursing care: the patient’s perspective | Leinonen et al. | Quality of intraoperative nursing care as evaluated by patients (biological–physiological, experiential, cognitive, functional, ethical, nursing professionals’ characteristics/competence, and environmental dimensions); patient satisfaction with intraoperative care | Intraoperative nursing care provided by operating room nurses (including keeping the patient warm, information provision, teamwork, privacy, and environmental management) | Surgical patients at a Finnish university hospital (n = 246 in hospital; n = 158 at home) | Structured questionnaire with seven quality dimensions, measured using visual analogue scales (100 mm) and structured multiple-choice items (Cronbach’s α = 0.47–0.83 in hospital; 0.51–0.79 at home) | End of hospital stay and approximately one month after surgery (at home) |
| **36** | Effect of preoperative forced-air warming on postoperative temperature and postanesthesia care unit length of stay | Fettes et al. | Postoperative body temperature (preoperative, on exiting preoperative area, and on PACU admission); PACU length of stay | Preoperative forced-air warming applied by perioperative nurses (blanket at 100°F/37.8°C, medium setting, approximately one hour before surgery); temperature monitoring across perioperative phases | Surgical patients (ASA I–III) at a community hospital in the United States (N = 128; warming n = 54, no warming n = 74) | Temporal artery-scanning thermometer (accurate to 0.1°C) for body temperature; recorded PACU length of stay | Preoperatively, on exiting the preoperative area, and on PACU admission (immediate postoperative/PACU phase) |
| **37** | Application of operating room nursing intervention to incision infection of patients undergoing gastrointestinal surgery can reduce complications and improve gastrointestinal function | Wang et al. | Incision infection signs (swelling, pain, secretion); wound healing grade (A/B/C); gastrointestinal function recovery (anal exhaust, first defecation, gastric tube removal times); postoperative complications; nursing satisfaction | Operating room nursing intervention (preoperative preparation and patient education, strengthened operating room management and asepsis, and meticulous incision care) | Patients undergoing gastrointestinal surgery in China (N = 340; operating room nursing n = 170, conventional nursing n = 170) | Clinical records of incision signs, wound healing grade, and gastrointestinal function times; complication incidence; nursing satisfaction rating scale (content reliability index 0.79) | Postoperative period (several days to one week; short- to mid-term) |

Note: OR, operating room; PHRCS, Perianesthesia Comfort Scale; SSI, Surgical Site Infection; HIPEC, Hyperthermic Intraperitoneal Chemotherapy; POD, Postoperative Day; EORTC IN-PATSAT32**,** EORTC In-Patient Satisfaction with Care Questionnaire
